# Aurora kinase protein family in *Trypanosoma cruzi*: Novel role of an AUK-B homologue in kinetoplast replication

**DOI:** 10.1371/journal.pntd.0007256

**Published:** 2019-03-21

**Authors:** Matías Fassolari, Guillermo D. Alonso

**Affiliations:** 1 Laboratorio de señalización y mecanismos adaptativos en tripanosomátidos, Instituto de Investigaciones en Ingeniería Genética y Biología Molecular “Dr. Héctor N. Torres”, Buenos Aires, Argentina; 2 Departamento de Fisiología, Biología Molecular y Celular, Facultad de Ciencias Exactas y Naturales, Universidad de Buenos Aires, Buenos Aires, Argentina; Instituto de Investigaciones Biotecnológicas, ARGENTINA

## Abstract

Aurora kinases constitute a family of enzymes that play a key role during metazoan cells division, being involved in events like centrosome maturation and division, chromatin condensation, mitotic spindle assembly, control of kinetochore-microtubule attachments, and cytokinesis initiation. In this work, three Aurora kinase homologues were identified in *Trypanosoma cruzi* (TcAUK1, -2 and -3), a protozoan parasite of the Kinetoplastida Class. The genomic organization of these enzymes was fully analyzed, demonstrating that TcAUK1 is a single-copy gene, TcAUK2 coding sequence is present in two different forms (short and long) and TcAUK3 is a multi-copy gene. The three TcAUK genes are actively expressed in the different life cycle forms of *T*. *cruzi* (amastigotes, trypomastigotes and epimastigotes). TcAUK1 showed a changing localization along the cell cycle of the proliferating epimastigote form: at interphase it is located at the extremes of the kinetoplast while in mitosis it is detected at the cell nucleus, in close association with the mitotic spindle. Overexpression of TcAUK1 in epimastigotes leaded to a delay in the G2/M phases of the cell cycle due a retarded beginning of kinetoplast duplication. By immunofluorescence, we found that when it was overexpressed TcAUK1 lost its localization at the extremes of the kinetoplast during interphase, being observed inside the cell nucleus throughout the entire cell cycle. In summary, TcAUK1 appears to be a functional homologue of human Aurora B kinase, as it is related to mitotic spindle assembling and chromosome segregation. Moreover, TcAUK1 also seems to play a role during the initiation of kinetoplast duplication, a novel role described for this protein.

## Author summary

The cell cycle is a complex and highly regulated cellular process in which different checkpoints are coordinated and a unique pattern of protein activities is present. In trypanosomatids, this process is even more complex because of the presence of the kinetoplast, a network of circular DNA inside a large mitochondrion that coordinates its division and segregation with the nuclear mitosis. Aurora kinases comprise a family of enzymes that regulate key steps during the cell cycle by varying their subcellular localization. In this work, we identified three Aurora kinase genes (TcAUK1, -2 and -3) in *T*. *cruzi*, the agent of Chagas disease, and established that TcAUK1 has the typical behavior of a mammalian Aurora B kinase, localizing inside the nucleus and associating with the mitotic spindle during mitosis. On the other hand, during interphase TcAUK1, localizes at each side of the kinetoplast, showing a novel localization that has not been described before. Finally, here we present evidence for the relationship between the novel localization of TcAUK1 and its participation in kinetoplast division.

## Introduction

Cell cycle in eukaryotic cells involves the sequential transition between G1, S, G2 and M phases and this progression is tightly regulated by protein kinases and phosphatases [1]. During the M phase, the cell divides its nucleus to originate two daughter cells with the same genetic content. In this event, Aurora kinase proteins play a crucial role. The Aurora kinase family of proteins presents a variable number of members among different organisms. While yeasts present a single Aurora kinase gene [2,3], organisms like *C*. *elegans* and *D*. *melanogaster* have two genes [4–7], whereas in vertebrates three Aurora kinase proteins are present [8]. In this last group, the three members of the Aurora kinase family are named Aurora-A, -B and -C and each protein plays specific functions during the cell cycle. In organisms with a single Aurora kinase gene, the encoded protein combines the function of both Aurora-A and -B, whereas in organisms with two Aurora proteins, one behaves as an Aurora-A while the other has functions similar to Aurora-B. In humans, Aurora-C is expressed in germinal cell lines and its function has not been elucidated yet. A distinctive feature of all Aurora proteins is that they change their cellular location during mitosis progression, according to their different roles. Aurora-A, the so-called Polar Aurora, is involved in centrosome maturation/migration and bipolar spindle formation/stabilization [9] and therefore, it is found in the neighborhood of the dividing centrosome in early mitosis, after which it moves with each of duplicated centrosome to opposite extremes of the cell, where the spindle poles locate during G2 phase [10]. Aurora-B is named Equatorial Aurora because it locates in the mid-plane of the cell during mitosis (metaphase) and, as nuclear division proceeds, it is tightly associated with segregating chromosomes. This Aurora forms the Chromosomal Passenger Complex (CPC) with other three proteins (INCENP, Survivin and Borealin). As part of this complex, Aurora-B promotes chromatin condensation in prophase through phosphorylation of histone H3 at Ser10 [11]. In metaphase, it participates at the Spindle Checkpoint to ensure correct chromosome segregation during anaphase [12]. Finally, in cytokinesis the CPC settles in the cell midzone and participates in the formation of the contractile ring and the cleavage furrow [13].

*Trypanosoma cruzi* is a protozoan of the Kinetoplastida order and is the etiological agent of the American Trypanosomiasis, also known as Chagas disease. This disease is endemic in Latin America but in the last decades, due to the increasing migratory flux, a growing number of infections have been detected in non-endemic countries like the United States and Spain [14]. The complex life cycle of *T*. *cruzi* involves different forms: the epimastigotes and metacyclic trypomastigotes are present in the insect vector, and the amastigotes and bloodstream trypomastigotes are found in the vertebrate host. All these forms contain a single flagellum emerging from the basal body, a nucleus and a mitochondrion carrying the DNA complex known as kinetoplast. During cell division, all these organelles are replicated and segregated into two daughter cells in a synchronized manner. Moreover, both the existing and newly synthesized basal bodies physically interact with the duplicating kinetoplast and drive its division [15], meaning that the synchronization is due in part to a physical contact between these organelles. During *T*. *cruzi* mitosis, contrary to what is observed in other organisms, the nuclear membrane remains intact and the chromosomes do not condensate. Despite this, the formation of a mitotic spindle inside the nucleus has been described [16] and it is well known that chromosome segregation is mediated by a microtubules-dependent mechanism [17]. While most eukaryotic cells contain many mitochondria with separate copies of circular DNA molecules, kinetoplastids have a single mitochondrion with a genome in which DNA molecules are physically interlocked forming a big network, the so-called kinetoplastid DNA (kDNA). These DNA molecules consist of two type of circles: the larger maxicircles that are the equivalent of the mitochondrial genome in other organisms and are present as several dozen identical copies per cell, and the smaller minicircles that codified for guide RNAs (gRNAs) and are in thousands copies per cell. During cell division, this DNA does not only need to be duplicated but a well-orchestrated de-concatenation and segregation, driven by the recently duplicated flagella basal bodies, needs to take place [18]. The complexity of the mitochondrion genome adds for a particular kDNA S-phase besides from the classical G1, S and G2/M cell cycle stages. Although contributions have been made by different authors to the knowledge about the kDNA replication mechanism [19], many of the molecular players involved in the later steps of this process remain obscure, mainly the ones involved in kDNA division and segregation.

Aurora kinase genes have been identified and described in different protozoan organisms. In *Leishmania major*, a single Aurora gene (Lmairk) has been reported but its function has not been studied yet [20]. In the apicomplexa *Plasmodium falciparum*, three Aurora genes have been described (Pfark -1, -2 and -3) Pfark-1 was defined based on its subcellular localization as the classic Aurora gene present in other organism, whereas the remaining two Pfark proteins seem to be involved in cellular processes exclusive of this organism [21]. Davids and coworkers found a single Aurora gene in the biflagellate *Giardia lamblia* [22], showing that this protein adopts a cellular localization similar to mammalian Aurora-A and Aurora-B and is involved in microtubules dynamics reorganization at mitosis and interphase. In *Trypanosoma brucei* three Aurora genes were identified by Wang and coworkers but gene silencing experiments demonstrated that only TbAUK1 is a functional gene, at least in the procyclic form [23]. This protein is involved in mitotic spindle assembling and it was defined by the authors as a human Aurora-B ortholog. Later, these same authors showed that TbAUK1 silencing in the bloodstream form leads to failure to conclude cytokinesis and cell shape alteration, both effects associated to a microtubule filaments disruption [24]. TbAUK1 seems to be associated to others proteins conforming a complex like the CPC of mammals and, as with Aurora B, the proteins of this complex affect TbAUK1 localization and function [25].

A comparative analysis of the kinomes of three pathogenic kinetoplastids—including *T*. *cruzi–*by Parsons and co-workers reported the presence of several kinases normally associated to roles in cell division, the Aurora kinases being part of this group [26]. In this work we report the initial characterization of three Aurora kinase proteins in *T*. *cruzi* (TcAUK1, TcAUK2 and TcAUK3). In addition, by a detailed analysis of TcAUK1 localization, we have found that this protein shows the canonical behavior of a chromosome passenger protein, being associated with the mitotic spindle during nuclear division. Furthermore, we detected that during interphase, TcAUK1 is located at both sides of the kinetoplast. Finally, we report that TcAUK1 overexpression in epimastigote forms causes a delayed G2-M transition, presumably by affecting the onset of kinetoplast duplication.

## Materials and methods

### Chemicals and reagents

Radio chemicals were purchased from PerkinElmer Life Sciences, and restriction endonucleases were from New England Biolabs, Beverly, MA. Bacto-tryptose, yeast nitrogen base, and liver infusion were from Difco. All other reagents were purchased from Sigma.

### Data mining and bioinformatics analyses

The gene sequences corresponding to TbAUK1 (Tb927.11.8220), TbAUK2 (Tb927.3.3920) and TbAUK3 (Tb927.9.1670) were used to screen *T*. *cruzi* sequences in TryTrip database using BlastN algorithm. Pairwise alignment and motif search were performed on high-scored targets by EMBL-EBI tools [27] and Pfam [28], as well as manual inspection. Multiple sequence alignment was performed in MEGA 5 software [29] with ClustalW algorithm and visualized with BioEdit software [30].

### *T*. *cruzi* epimastigotes culture and growth analysis

*T*. *cruzi* epimastigote of CL Brener strain were grown at 28°C with 5% CO_2_ in LIT medium [5 g.l^-1^ liver infusion, 5 g.l^-1^ Bacto-tryptose, 68 mM NaCl, 5.3 mM KCl, 22 mM Na_2_PO_4_, 0.2% (w/v) glucose, 0.002% (w/v) hemin] containing 10% v/v fetal bovine serum (NATOCOR, Argentina), 100 units.ml^-1^ penicillin and 100 μg.l^-1^ streptomycin. Cell density was maintained between 1x10^6^ and 1x10^8^ cells.ml^-1^ sub-culturing parasites every 6–7 days. For growth curve determinations, a sample of culture supernatant was taken and swimming epimastigotes were fixed by incubation in 4% formaldehyde in PBS for 5 min at room temperature. Cell density was determined by counting at least three independent cultures in an hemocytometer. Specific growth rate (μ, expressed as h^-1^) was estimated by the slope of the graphic “Ln of culture cell density” vs “culture time” (h). Cells duplication time (DT) was calculated according to the formula:

$$DT=\frac{ln2}{\mu }$$

### Vero cells infection

*Cercopithecus aethiops* (green monkey) Vero cells (ATCC CCL-81) were cultured at 37°C and 5% CO_2_ supplied in Minimum Eagle Medium (MEM, Gibco) supplemented with 10% fetal bovine serum (HyClone), 2 mM L-Glutamine (Sigma), 100 units.ml^-1^ penicillin and 100 μg.l^-1^ streptomycin. For the obtention of *T*. *cruzi* trypomastigotes and amastigotes Vero cells were infected with trypomastigotes (1:50 ratio) 24 h after being plated and maintained in MEM supplemented with 3% FBS. Trypomastigotes in culture supernatant were harvested by centrifugation and processed as needed. Amastigotes were collected from 10–11 day old cultures from the supernatant (90% or higher amastigotes/trypomastigotes ratio), centrifuged, and processed as needed.

### Molecular cloning of TcAUKs

Based on the sequences of Aurora kinases homologs found in *T*. *cruzi* database, specific primers were designed: TcAUK1 (TcAUK1-NcoI-fwd 5´-CCATGGTGAGTGCGGCGGAGGGCGGCCAA-3´ and TcAUK1-XhoI-rev 5´-CTCGAGGTTCTCCTTTCCGCCCGAGAAGT-3´), TcAUK2 (TcAUK2-BamHI-fwd 5´-GGATCCGCAGCACCACAACTTGAGTTCC-3´ and AUK2-XhoI-rev 5´- CTCGAGCTTCTTCTTCTTCTTCTCCCCATTT-3´), TcAUK3 (AUK3-NcoI-fwd 5´- CCATGGTGTGGTCGCTGGATGACTTTGAT-3´ and AUK3-XhoI-rev 5´-CTCGAGTAAATTCTCTGCCGCATCAACCGT-3´). Polymerase Chain Reaction (PCR) was performed in a PTC-150 MiniCycler (MJ Research). For this, genomic DNA of *T*. *cruzi* was isolated as described previously [31] and gene amplification was performed by using a high fidelity DNA polymerase (Herculase II, Stratagene). The thermal cycling conditions were specific for each TcAUK gene. Amplification products were gel-purified, subcloned into pGEM-T Easy vector (Promega), transformed into *E*. *coli* DH5α competent cells and both strands were sequenced (Macrogen, Korea).

### Genomic organization analysis

Genomic DNA of epimastigote forms was digested (approx. 30 μg) overnight with 30 units of the indicated restriction enzyme (New England Biolabs). After digestion, DNA was SpeedVac concentrated (Jouan RCT 60 Refrigerated Cold Trap) and electrophoresed for 8–12 h in 0.8% agarose gel (1 V/cm) and was then denatured, neutralized and transferred onto nylon membrane (GeneScreen, Perkin Elmer) for Southern blot analysis [32]. For this, specific radiolabeled probes were generated by primer extension using full-length TcAUK genes and [α-^32^P]-dCTP (NEBlot kit, New England Biolabs). dCTP radiolabeled probes were then purified with MicroSpin G-50 columns (GE Healthcare) and heat denaturalized before proceeding with hybridization. Probes were hybridized at 65°C (overnight) and washed at 65°C using 2x SSC, 1xSSC and 0.5x SSC with 0.1% SDS sequentially to remove excess of probe. Blot was developed by exposing membrane to Phosphoimager Storm system (Pharmacia-Biotech).

To perform Pulsed Field Gel Electrophoresis, CL Brener exponentially growing epimastigotes were washed with PBS-Glucose 2%, suspended in PBS and mixed with one volume of low melting point agarose 1.4% in PBS. After polymerization, the agarose plug was incubated with LIDS buffer (1% Lauryl Sulfate Lithium Salt, 10 mM Tris-HCl pH8.0, 0.1 M EDTA) for 48 h at 37°C. Afterwards the blocks were washed 6 times with NDS 0.2% buffer (0.2% N-Lauroylsarcosine-Sodium Salt, 0.1 M EDTA, 2 mM Tris base) and, before running the agarose gel, they were equilibrated in TE buffer pH 8,0. Gel electrophoresis was performed at 16°C in three steps: 1) 3 V/cm changing periods every 90–200 sec during 30 h; 2) 3 V/cm changing periods every 200–400 sec during 30 h; 3) 2.7 V/cm changing periods every 400–700 sec during 24 h. After electrophoresis, DNA was transferred to a nylon membrane and hybridized with TcAUKs probes as described above for Southern blot analysis.

### Gene expression analysis

Total RNA was isolated from epimastigotes, trypomastigotes and amastigotes forms using Trizol according to the manufacturer’s protocol (Invitrogen). cDNA was obtained from mRNA by Transcriptor First Strand cDNA Synthesis Kit (Roche) using oligo(dT)_18_ primer, following the supplier´s instructions. These cDNA samples were used to amplify a fragment of TcAUKs genes and the housekeeping Actin gene (TcCLB.510945.30). For all TcAUKs targets the same forward Tc-SL primer was used (5´-AACGCTATTATTGATACAGTTTC-3´) whereas a specific reverse primer for each one was designed: TcAUK1-RT-rev (5´-CCACCCAAAGTCTGCCAACTTA-3´), TcAUK2-RT-rev (5´-AGCGTGCGGTGAACGTTGATCT-3´) and TcAUK3-RT-rev (5´-AATCCATCGTGCCGCAAAGCGT-3´). In the case of the housekeeping actin gene the primers used were: TcActin-fwd (5´-ATGATCATCGTGGACTTTGGGT-3´) and TcActin-rev (5´-TTCCGCTTGGGTGTGAACAGC-3´). The PCR reactions included an initial denaturalization step at 95°C for 2 min, followed by 35 cycles at 95°C for 1 min, annealing at 60°C for 45 sec, extension at 72°C for 45 sec and a final extension at 72°C for 5 min. Amplification products were visualized by electrophoresis on 1.5% agarose gel. Then, they were gel extracted, subcloned into pGEM-T Easy vector (Promega) and sequenced (Macrogen, Korea).

For western blot analysis cells were suspended in lysis buffer (50 mM Tris-HCl pH 8.0, 1 mM EDTA, 1 mM DTT, 0.1% Triton X-100, 1% NP-40, 1 mM PMSF and 1 μg. ml^-1^ E-64) and lysed by freeze/thaw cycles in liquid nitrogen. The obtained lysate was centrifuged at 10,000 x *g* for 30 min and the pellet was discarded. Total protein concentration in the extracts (supernatant) was estimated by the Bradford quantification, and an aliquot containing 20–80 μg of proteins was loaded onto 12% (w/v) SDS-polyacrylamide gel, solved by electrophoresis as described by Laemmli [33] and electro-transferred to nitrocellulose membranes (Hybond-C, Amersham Pharmacia Biotech). The membranes were then blocked with 5% (w/v) non-fat milk suspension in TBS-Tween 0.05% for 2 h and TcAUK1 was detected with a rabbit antiserum to TcAUK1 (custom produced by GenScript Corporation against the peptide PRGKRMRGAADFSG, amino acids 292 to 305 of TcAUK1) and a goat antiserum to rabbit IgG HRP-labeled secondary antibody (PerkinElmer). Specific TcAUK1 signal was developed with the ECL Plus Western blotting detection system (PerkinElmer Life Sciences).

### Overexpression of TcAUK1 protein

The full-length coding sequence of TcAUK1 gene sub-cloned into pGEM-T Easy vector (see above) was isolated by digestion with EcoRI and XhoI nucleases and cloned into pTREX plasmid [34] digested with the same restriction enzymes. *T*. *cruzi* epimastigotes of CL Brener strain were electro-transfected with empty pTREX plasmid or the pTREX-TcAUK1 construct as described previously [34]. Stable transfectant pools were achieved after 60 days of treatment with 500 μg.ml^-1^ G418 (Gibco BRL, Carlsbad, CA). Once selection has finished, single clone cell cultures were obtained for pTREX-TcAUK1 transfectants by the limit-dilution cloning method. Transgenic condition of several clones was confirmed by Southern and Western blot analyses.

For the expression of the fusion protein TcAUK1-GFP, the coding sequence of TcAUK1 was amplified by PCR with primers TcAUK1-NcoI-fwd (5´-CCATGGTGAGTGCGGCGGAGGGCGGCCAA-3´) and TcAUK1-Rev-STOPLess-BamHI (5´-GGATCCGTTCTCCTTTCCGCCCGAGAAGTCC-3´). The amplification product was sublconed into pGEM-T Easy vector, isolated by digestion with EcoRI and BamHI endonucelases and cloned into pTEX-eGFP-TEV-HA-EEF plasmid (kindly donated by Dr. Leon A. Bouvier, Instituto de Investigaciones Biotecnológicas, IIB-INTECH) digested with the same restriction enzymes. *T*. *cruzi* epimastigotes of CL Brener strain were electro-transfected with this construct and localization of fusion protein TcAUK1-GFP was evaluated after 48 h by fluorescence microscopy.

### Immunolocalization

*T*. *cruzi* epimastigote and trypomastigote forms were harvested by centrifugation, washed once with PBS and allowed to adhere to poly-L-lysine coated coverslips. Vero cells cultured in 24 wells plate with a sterile coverslip, were infected and at different days after infection, culture medium was removed and cells were washed once with PBS previous to further processing. Parasites and infected Vero cells were fixed in 4% paraformaldehyde and washed twice with PBS. After been permeabilized with 0.2% Triton-X100 in PBS (PBT solution), cells were treated with blocking solution (1% BSA in PBS) for 1 h at room temperature. For TcAUK1 and mitotic spindle double staining, epimastigote forms were first incubated with a mix of the rabbit antiserum to TcAUK1 (1:200 dilution, GenScript Corporation) and the monoclonal mouse anti-β-tubulin KMX-1 antibody (1:400 dilution, Chemicon International) and then incubated with a mix of goat anti-rabbit IgG Alexa Fluor 488-labeled (1:500 dilution, Invitrogen) and goat anti-mouse IgG Alexa Fluor 594-labeled (1:500 dilution, Invitrogen). For TcAUK1 labeling in trypomastigote and amastigote, cells were first incubated with the rabbit antiserum to TcAUK1 and then with the secondary antibody goat anti-rabbit IgG Alexa Fluor 488-labeled. In all cases cells were incubated with the first antibody for 1 h at room temperature, then washed three times with PBT solution and finally incubated with the secondary antibody another hour at room temperature. After being washed three times with PBT solution, the slides were mounted in VectaShield mounting medium (Vector Labs) containing DAPI and examined with a fluorescence microscope (model BX41, Olympus). For actin filament staining, Vero cells were incubated for 10 min at room temperature with Rhodamine Phalloidin (1:1000 dilution, Invitrogen), washed and mounted as described previously for the secondary antibody. Images were processed with the ImageJ software [35].

### Cell cycle analysis

Synchronization of epimastigote forms of *T*. *cruzi* in G1/S of the cell cycle was achieved using hydroxyurea (HU). Cells in exponential growth phase were arrested by incubation with 15 mM of HU for 20–24 h and then released by washing twice with PBS and suspending the cells in culture medium. Cells continued to be cultured for 20 h; samples were taken at the indicated time points and processed as indicated. For flow cytometry analysis, 3x10^5^ cells were harvested by centrifugation, washed with PBS-EDTA 2 mM and fixed in 70% ethanol at -20°C for 30 min. Then, they were washed once with PBS and suspended in staining solution (69 μM propidium iodide, 38 mM citrate buffer pH 7.40, 0.2 mg.ml^-1^ RNase). The DNA content of propidium iodide-stained cells was analyzed with a fluorescence-activated cell sorting (FACSAria II) analytical flow cytometer (BD Biosciences). Percentages of cells at different phases of the cell cycle were evaluated by Cyflogic software. For microscopic observation of cell cycle progression, synchronized cells were processed as described for the immunolocalization assay, using KMX-1 antibody.

## Results

### Identification and in silico characterization of the Aurora Kinase family members in *Trypanosoma cruzi*

**Fig 1:**
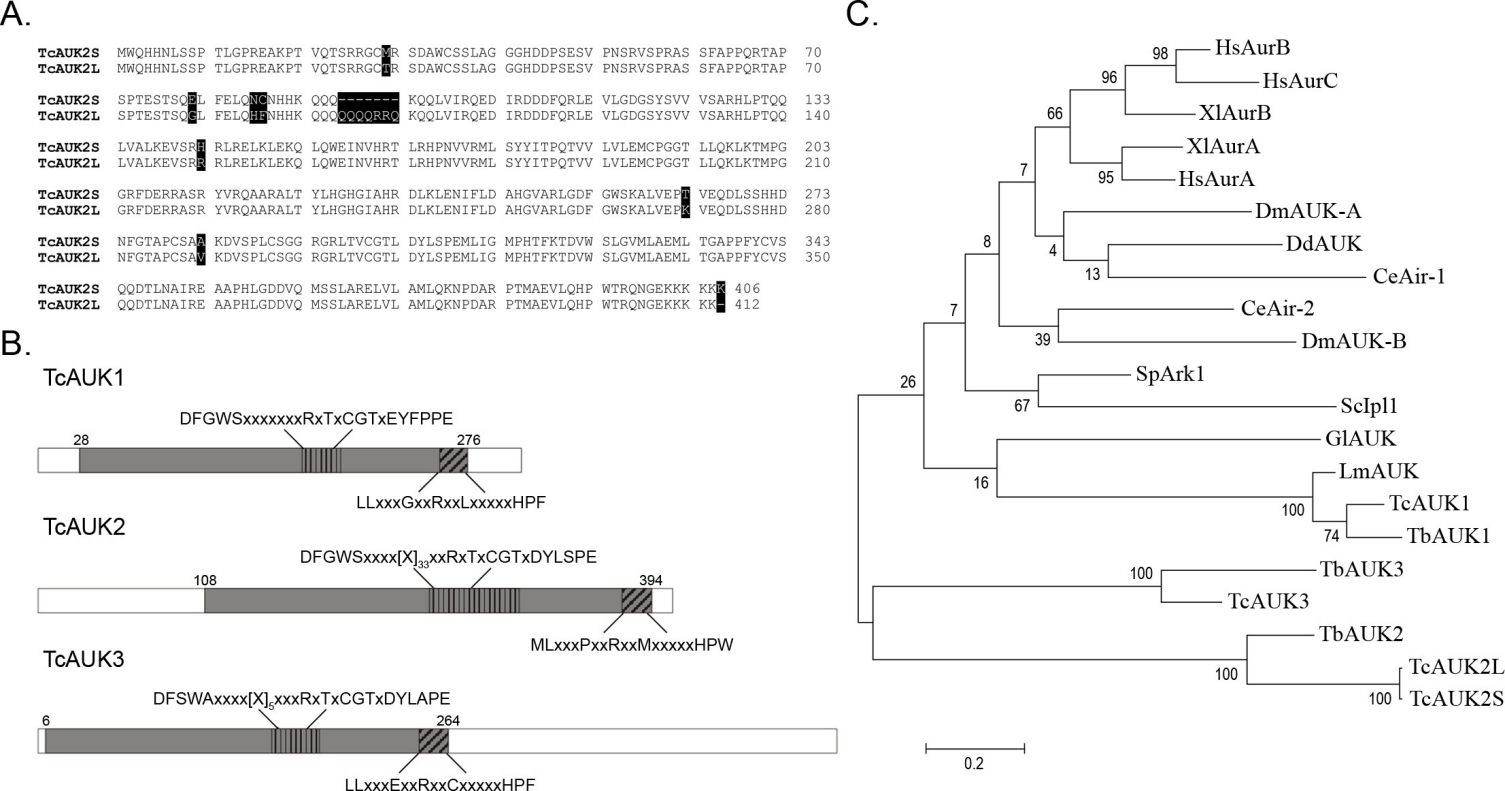
Sequence analysis of TcAUK proteins. **(A)** Pairwise alignment of deduced amino acid sequences of TcAUK2S and TcAUK2L highlighting the differences between them (black shadow). **(B)** Schematic representation of TcAUKs showing domain organization in TcAUK proteins. In plane grey is the kinase catalytic domain, including the highly conserved Activation loop (vertical black lines) and Destruction Box (sloping black lines). **(C)** Evolutionary tree constructed by the maximum parsimony method from a MSA of TcAUKs and aurora genes described for metazoans and protozoans. Hs *Homo sapiens*, Xl *Xenopus laevis*, Dm *Drosophila melanogaster*, Dd *Dictyostelium discoideum*, Ce *Caenorhabditis elegans*, Sp *Schizosaccharomyces pombe*, Sc *Saccharomyses cerevisiae*, Gl *Giardia lamblia*, Lm *Leishmania major*, Tb *Trypanosoma brucei*.

The protein sequences of *T*. *brucei* Aurora kinase genes (TbAUK1, TbAUK2 and TbAUK3) were used as baits to search for orthologue sequences in the *T*. *cruzi* genome database (http://tritrypdb.org/tritrypdb/). As this database has been made from sequencing the genome of CL Brener strain, a hybrid [36,37] that arose from two different lineages (Esmeraldo-like and Non-Esmeraldo-like haplotypes), we found several putative genes for each TbAUK. When TbAUK1 and TbAUK2 were used as query, two coding sequences (CDS) for each one were found, representing in both cases the same allele for the different haplotypes. In the case of TbAUK3, three CDS were detected, two of them corresponding to the Esmeraldo-like and the other one from the non-Esmeraldo-like haplotype. After an exhaustive sequence analysis, we established that both CDS related to TbAUK1 codify for a single amino acid sequence, and the same result was found with the three CDS related to TbAUK3. Nevertheless, the two CDS related to TbAUK2 showed conspicuous differences, including an insertion of 21 nucleotides. These identified sequences were named TcAUK1, TcAUK2 and TcAUK3, in accordance with the TbAUKs given names (for details see Table 1). In the case of TcAUK2, the two protein sequences were named TcAUK2S (short isoform) and TcAUK2L (long isoform with the 21 nucleotides insertion). Fig 1A shows the sequence alignment of TcAUK2S and TcAUK2L where the dissimilarities and the insertion of seven residues are highlighted. After establishing the CDS for each TcAUK, specific oligonucleotides were designed and used to amplify these genes, using genomic DNA of *T*. *cruzi* CL Brener strain as template. The obtained amplification products were then subcloned, sequenced and compared to nucleotide sequences found in the database. While sequenced TcAUK1 was identical to the gene found in the *T*. *cruzi* Genome Project database, TcAUK2 as well as TcAUK3 sequences showed some minor discrepancies. Particularly in the case of TcAUK2, the sequencing reactions confirms the presence of the variants TcAUK2S and TcAUK2L in the genome of the parasite. Once the final sequence of each gene was determined, they were annotated in GenBank under the following accession numbers: TcAUK1 EU494590.1, TcAUK2S EU494591.1, TcAUK2L EU494592.1, and TcAUK3 EU494593.1.

**Table 1:** Identification of TcAUKs CDS.

Template	CDS	Given ID	GenBank Accession number
TbAUK1 (Tb927.11.8220)	TcCLB.508817.80	TcAUK1	EU494590.1
	TcCLB.503799.40		
TbAUK2 (Tb927.3.3920)	TcCLB.503685.10	TcAUK2S	EU494591.1
	TcCLB.509999.110	TcAUK2L	EU494592.1
TbAUK3 (Tb927.9.1670	TcCLB.510349.80	TcAUK3	EU494593.1
	TcCLB.506715.10		
	TcCLB.504655.30		

A series of multiple sequence alignments (MSA) were performed to analyze the detected Aurora kinase genes of *T*. *cruzi*. A MSA of TcAUK proteins with the catalytic domain of human Protein kinase A (PKA) showed that most amino acids with key roles in the kinase activity of PKA are conserved in TcAUKs (S1 Fig). The most relevant is the presence of catalytic glutamic acid of PKA preceded by an Arginine, which is conserved in TcAUKs, allowing to classify them as members of the RD kinases group (S1 Fig, indicated as (1)). In a second MSA, TcAUKs deduced protein sequences were aligned with Aurora kinase proteins from model metazoans. The four TcAUKs present the two most characteristic domains of Aurora proteins: the *Activation loop* (**DFGWSxxxxxxRxTxCGTxDYLPPE**) and the *Destruction-box* (**LLxxxPxxRxxLxxxxxHPW**). The Threonine residue found in the highly conserved RxT motif from the Activation loop is phosphorylated in the active form of Aurora kinases enzymes (Fig 1B). Described in detail in human Aurora A protein, the 3D active site of this family of proteins involves the Activation loop and the Glycine Rich Loop [38]. In between them is the Hinge region, which along with other residues, forms a hydrophobic pocket where the purine ring of the ATP’s adenosine substrate is located. TcAUKs proteins also show a strong conservation of the main residues implicated in the folding of this hydrophobic pocket. The 3D structure of human Aurora kinase A in complex with ATPγS reported by Nowakowsky and co-workers [39] reveals that in its active state, the αC helix in the protein adopts a position that allows a salt bridge formation between residues Glu181 and Lys162. This salt bridge is essential for the catalytic activity of the enzyme, and it is very close to the β phosphate from de ATPγS ligand. The key residues are conserved in all the Aurora kinase proteins, including the TcAUKs. Finally, a phylogenetic analysis was carried out based on a third MSA of TcAUKs with Aurora kinases proteins of model metazoan and Aurora proteins described in other protozoans. The full-length amino acid sequences were aligned and a maximum parsimony tree was constructed (Fig 1C). As expected, the three TcAUKs group with its orthologues in *T*. *brucei*. On the other hand, the longer C-terminal sequences for TcAUK3 and a presence of a large inserted region in the Activation Loop of TcAUK2S and TcAUK2L make these proteins to group separately from the Aurora kinases of other species. Similarly, when a MSA was performed considering only the catalytic domain (S2 Fig) of these proteins, again TcAUK3 and both TcAUK2 grouped apart from the proteins of others organisms. Thereby, is TcAUK1 the one closest related to Aurora proteins of other protozoan and metazoans.

The complete analysis of the TcAUKs sequences show strong evidences that allow us to conclude that these genes most likely code for the *T*. *cruzi* aurora kinase functional orthologues.

### Genomic organization of TcAUKs genes

**Fig 2:**
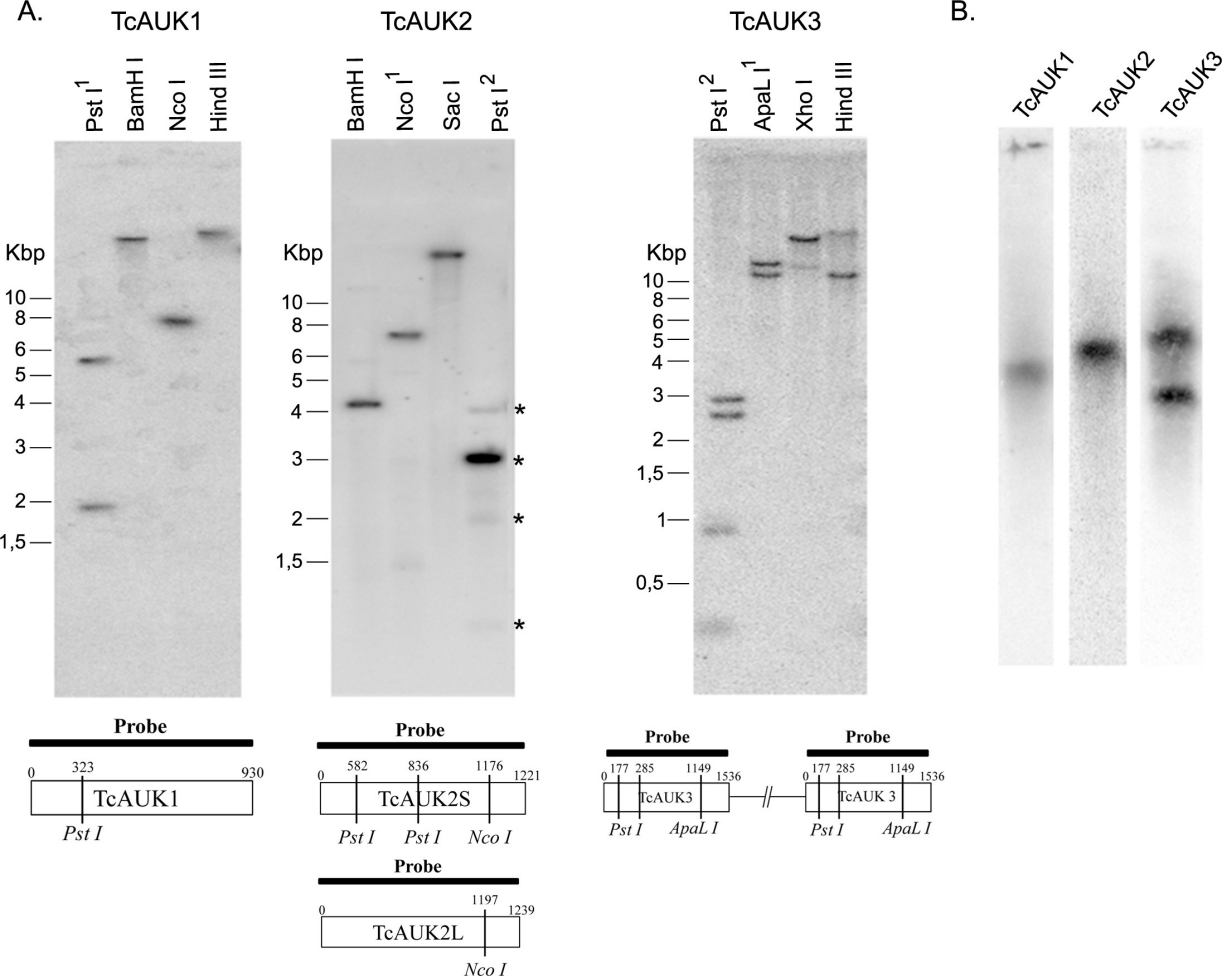
Genomic organization of TcAUKs genes. **(A)** Southern blot with TcAUKs probes and genomic DNA of *T*. *cruzi* digested with restriction endonucleases with target sites outside the TcAUK genes, and with one (1) or two (2) restriction site inside the sequence of the corresponding TcAUK gene. Asterisks at PstI line indicate the four obtained bands. **(B)** Pulsed Field Gel Electrophoresis (PFGE) of *T*. *cruzi* epimastigotes probed against each one of the TcAUK genes.

As described above, Aurora kinases from *T*. *cruzi* are represented in the database as more than one CDS. A detailed analysis of the information found in the database suggests that the CDS retrieved for TcAUK1 and TcAUK2 correspond to allelic variants of single copy genes. For TAUK3 the information in the database it is not conclusive about the number of CDSs and their corresponding genomic localization. A Southern blot analysis with specific probes for each TcAUK was performed on genomic DNA of *T*. *cruzi* CL Brener strain to experimentally determine the number of copies (Fig 2A). When TcAUK1 or TcAUK2 probes were used, the results confirm that both are single copy genes. Notably, the pattern obtained with the TcAUKs probe on genomic DNA digested using PstI demonstrated the existence of the TcAUK2S and TcAUK2L variants. Endonuclease PstI has two recognition sites within the sequence of the TcAUK2S variant but none inside the TcAUK2L. Consistent with the presence of both variants of TcAUK2, the total number of bands observed in the Southern blot were four: three corresponding to TcAUK2S and one for TcAUK2L (Fig 2A, TcAUK2 panel, line Pst1, asterisks). The Southern blot results obtained using a probe against TcAUK3 showed that when restriction enzymes without recognition sites within the protein coding sequence were used two bands were observed, indicating the presence of more than one copy of this gene. However, when restriction enzymes with one digestion site within the sequence were employed, there was a difference with the expected number of bands. The analysis of the CDS of TcAUK3 and their surrounding sequence obtained from TriTrypDB showed that TcAUK3 is present in three copies per haploid genome with two CDS in a tandem arrange on chromosome TcChr-33 and the third CDS in another contig without chromosome assignation. The tandem CDSs show high sequence identity on its flanking regions (98% identity), indicating the occurrence of a duplication event involving a large chromosomic region in which TcAUK3 is included. Given the difficult interpretation of this Southern blot results, Pulsed Field Gel Electrophoresis (PFGE) to separate intact chromosomes followed by hybridization with a TcAUK3 probe (Fig 2B) was carried out. Probes for TcAUK1 and TcAUK2 were included in this experiment to confirm the Southern blot results. While only one band was observed for TcAUK1 and TcAUK2, supporting results that indicated these are single copy genes, two bands were detected for TcAUK3, confirming the presence of more than one CDS located in different chromosomes. This result, together with the restriction profile observed in the Southern blot analysis, allows us to conclude that TcAUK3 gene is present in three copies per haploid genome of *T*. *cruzi* CL Brener strain. Additionally, in a recent publication [40] Dr. Robello and collaborators present the analysis of the genome sequences of two *T*. *cruzi* clones TCC (TcVI) and Dm28c (TcI), determined by PacBio Single Molecular Real-Time technology. The assemblies obtained with this technology permitted accurately estimate gene copy numbers. Analyzing this improved genome sequence, it was possible to confirm the presence of three copies of TcAUK3 gene in Dm28c: two at scaffold 196 and one at scaffold 24 (Dr. Robello, personal communication).

### TcAUK genes expression in *T*. *cruzi* life cycle

**Fig 3:**
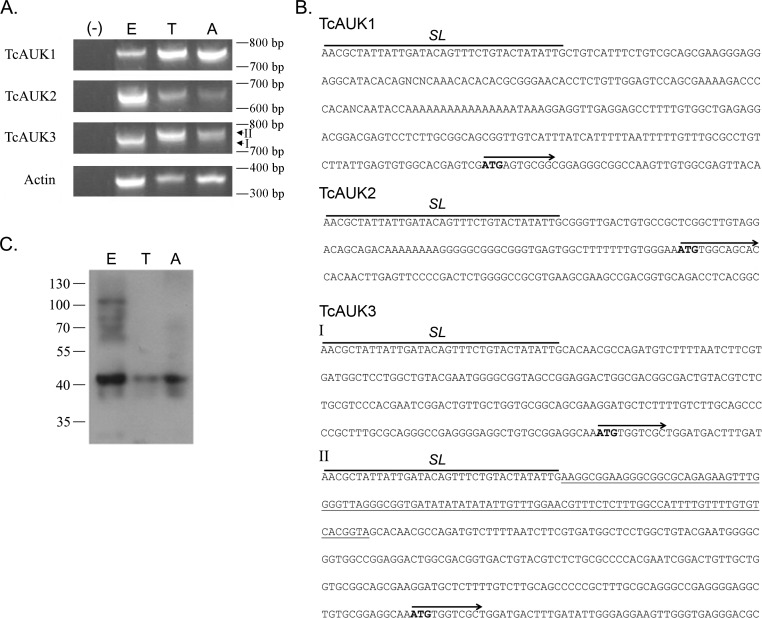
Expression of TcAUKs genes. **(A)** RT-PCR of the three TcAUKs and Actin (housekeeping control) in epimastigotes (E), trypomastigotes (T) and amastigotes (A) of *T*. *cruzi*. (-): PCR negative control. I and II indicate the specific amplification products of similar length obtained for TcAUK3 **(B)** Nucleotide sequence of the 5´ Untranslated Region of each TcAUK mRNA. The Spliced Leader sequence and the starting ATG codon followed by the initial sequence of the genes (CDS) are indicated. I and II reference to the bands indicated in (A). Underlined in TcAUK3 is the region of the 5´ UTR absent in one of the two amplification products. **(C)** Immunoblot of TcAUK1 in total cell extracts from Epimastigotes (E), Trypomastigotes (T) and Amastigotes (A).

After TcAUKs genes were identified, their expression through parasite life cycle was evaluated. The presence of the different TcAUK transcripts was evaluated by RT-PCR in epimastigote, trypomastigote and amastigote forms. Specific reverse primers for each TcAUK were used, while a primer corresponding to the Splice Leader (SL) region was used in all three cases. Amplification products were detected for each TcAUKs in all the three parasite forms (Fig 3A). In order to confirm their identity all amplification products were subjected to sequencing reactions. Sequence data obtained from TcAUK2 RT-PCR products confirms that both variants of this gene–TcAUK2S and TcAUK2L–are transcribed. Furthermore, 5´UTR region shows 100% of identity between variants, supporting the hypothesis that these two forms correspond to alleles inherited from two different parental lineages. In the case of TcAUK3, two specific amplification products of similar length (Fig 3A, I and II) were obtained. Sequencing showed that there a 100 bp stretch in the 5´UTR present in only one of the transcripts (Fig 3B, TcAUK3, II, underline), while the rest of the sequence had 100% identity with the shorter product (Fig 3B, TcAUK3). Thereby, the existence of two amplification products for TcAUK3 with differences in the 5´UTR agrees with what was observed in the Southern blot and the PFGE analysis, indicating the presence of more than one gene copy per haploid genome.

The phylogenetic tree in Fig 1C suggests that TcAUK1 shows greater similarity to metazoan Aurora genes than the rest of the TcAUKs. Since TbAUK1 from *T*. *brucei* is regarded as the protozoan counterpart of human Aurora B [23], we focused on unravelling the role of TcAUK1 in *T*. *cruzi* biology. Taking into account that in *T*. *cruzi* most of the regulation of protein expression occurs at the post-transcriptional level, the existence of TcAUK1 protein was evaluated by immunoblotting using a specific polyclonal antiserum against this protein. TcAUK1 protein was detected in whole cell extracts from epimastigotes, trypomastigotes and amastigotes (Fig 3C), hence consistent with mRNA expression data.

### TcAUK1 localization during cell cycle

**Fig 4:**
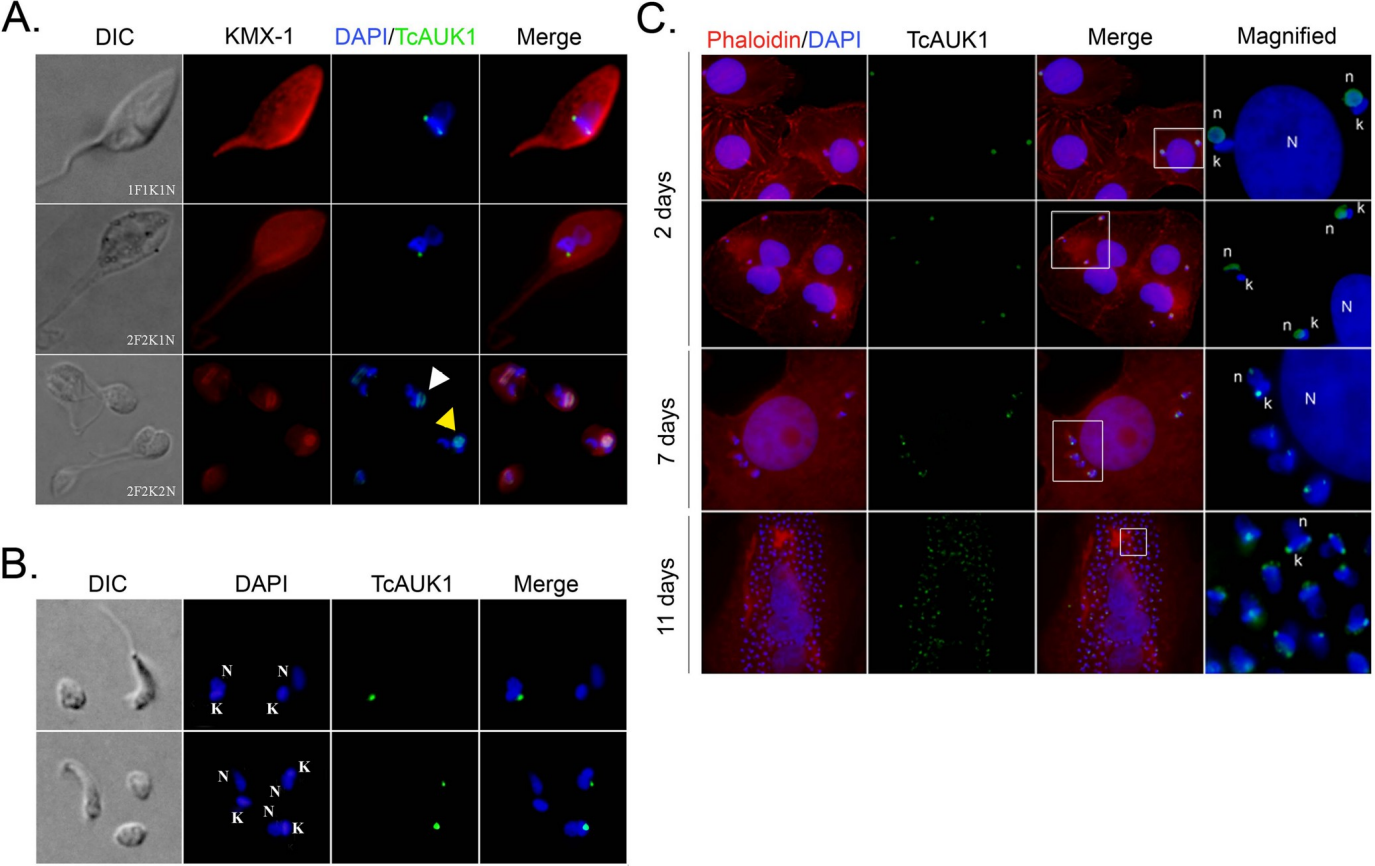
TcAUK1 localization in the different forms of *T*. *cruzi*. **(A)** Epimastigote forms at different points of the cell cycle (1F1K1N, 2F2K1N, 2F2K2N) were co-immunostained with rabbit antiserum to TcAUK1 and mouse KMX-1 for TcAUK1 and mitotic spindle, respectively. Yellow arrowhead indicates cells where the nuclei have not segregated yet, and white arrowhead points cells where both nuclei are segregating and **(B)** Amastigote and Trypomastigote forms isolated from culture supernatants were immunostained with rabbit antiserum to TcAUK1. **(C)** Infected Vero cells were stained with rhodamine-conjugated phalloidin for actin filaments and intracellular parasites were immunostained with rabbit antiserum to TcAUK1. In all cases, to dye DNA structures–nucleus (n or N) and kinetoplast (k)–cells were counterstained with DAPI. White squares point the magnified regions.

A main characteristic of Aurora kinase proteins is their dynamic redistribution during cell cycle. Human Aurora-B localization during mitosis has been well documented, at the beginning of the mitotic process it is dispersed in the nucleus, after which becomes concentrated in the nuclear midzone and migrates with chromatids to the cell poles, to finally locate in the constriction ring during cytokinesis. To address if TcAUK1 shares this dynamic localization during cell division, this protein was followed along cell cycle in epimastigote forms by immunostaining. DNA of the nucleus and kinetoplast were detected by DAPI staining while mitotic spindle microtubules were labeled using KMX-1 monoclonal antibody. This allowed to identify the different cell cycle stages in an asynchronous population based on the determination of the number of flagella (F), nuclei (N) and kinetoplasts (K), three organelles that duplicate in a tightly coordinate manner. In cells at interphase (1F1K1N), TcAUK1 was observed as a punctuated pattern localized in both extremes of the kinetoplast (Fig 4A, upper panel). The same localization was observed at late G2 when cells have already duplicated their flagellum and kinetoplast (2F2K1N) (Fig 4A middle row panels). In these cells, the two kinetoplasts are arranged in an anteroposterior alignment indicating the beginning of kinetoplasts segregation. During mitosis (2F2K2N), when both kinetoplasts are moving in opposite directions, TcAUK1 was detected inside the nucleus. In cells where the nuclei have not segregated yet, TcAUK1 adopted a circular conformation (Fig 4A lower row panels, yellow arrowhead), whereas in cells where both nuclei were segregating TcAUK1 was observed as an elongated structure (Fig 4A lower row panels, white arrowheads). Furthermore, TcAUK1 adopted the same configuration as the mitotic spindle, as shown by microtubule staining, revealing a close association of TcAUK1 with this structure.

To study TcAUK1 localization in the other forms of *T*. *cruzi*, trypomastigote and amastigote forms were collected from culture supernatant of infected Vero cells and subjected to immunostaining with TcAUK1 antiserum (Fig 4B). In amastigotes, TcAUK1 appeared as a single focus close to the kinetoplast, whereas in trypomastigotes no TcAUK1 signal was detected. Given that amastigotes represent the intracellular replicative form of *T*. *cruzi* we prompted to study TcAUK1 localization in *Trypanosoma cruzi* during the progression of cell infections, using Vero as a host cell. In Fig 4C it can be observed that at day 2 after infection, both amastigotes and trypomastigotes were present inside the cytoplasm of Vero cells. Surprisingly, here TcAUK1 was detected not only in amastigotes but also in trypomastigotes and in both cases located inside the parasite nucleus (Fig 4C, 2 days). At days 7 and 11 post-infection, only amastigotes could be observed inside Vero cells, and TcAUK1 was detected as two foci located one at each side of the kinetoplast, similar to what was observed in epimastigote forms during interphase (Fig 4C, 7 days and 11 days).

**Fig 5:**
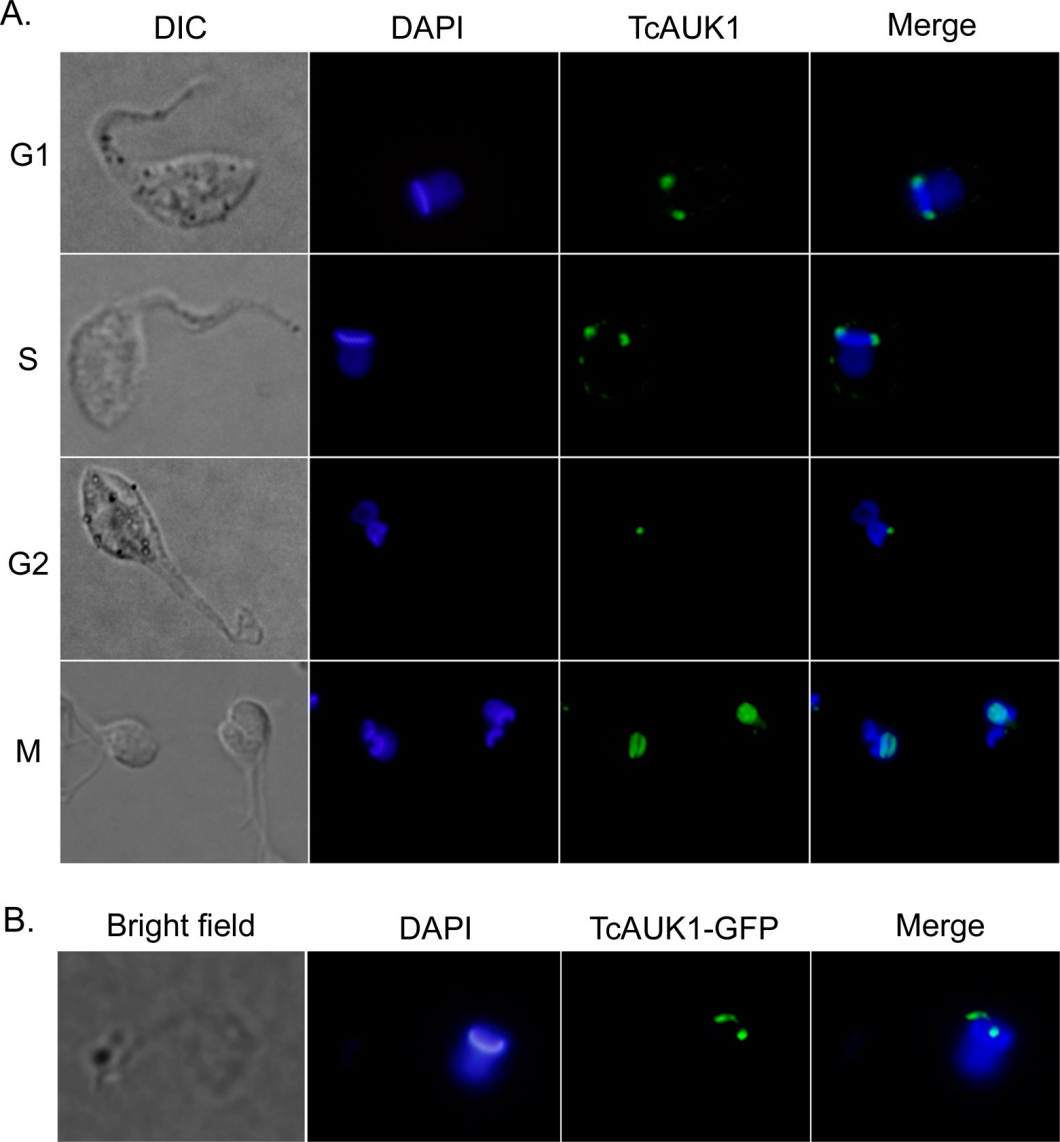
TcAUK1 localization in epimastigote forms. **(A)** Epimastigote forms were cell cycle synchronized with HU and the indicated cell cycle phases confirmed by flow cytometry. TcAUK1 (rabbit antiserum to TcAUK1) and nucleus/kinetoplast (DAPI). **(B)** TcAUK1 coding sequence was cloned into pTEXeGFP expression vector and epimastigotes were transfected. Shortly after transfection (24–48 hs), the localization of the fusion protein was evaluated by fluorescence microscopy.

The above observations indicate that during the cell cycle TcAUK1 shows a changing localization. To accurate define each cell cycle phase, we synchronized epimastigote forms by hydroxyurea (HU) treatment. After release, samples were taken at different time points for flow cytometry and for determine TcAUK1 localization by immunofluorescence. Fig 5 shows that in G1 and S phases, when the cell has 1 nucleus and 1 kinetoplast, TcAUK1 was located at both extremes of the kinetoplast. At the moment that the kinetoplast has duplicated in G2 phase, TcAUK1 was still close to the kinetoplast but it moved out from the extremes and appeared as a single point. Finally, in M phase TcAUK1 was detected inside the dividing nucleus. According to what was observed in Fig 4A, and considering the role that Aurora proteins from other organisms play, during M phase TcAUK1 could be involved in mitotic spindle dynamics and chromatin segregation. Nevertheless, the localization of TcAUK1 in the proximity of the kinetoplast during interphase leads to ask about the role that it is playing in that location.

### Phenotypic analysis of TcAUK1 overexpressing epimastigotes

**Fig 6:**
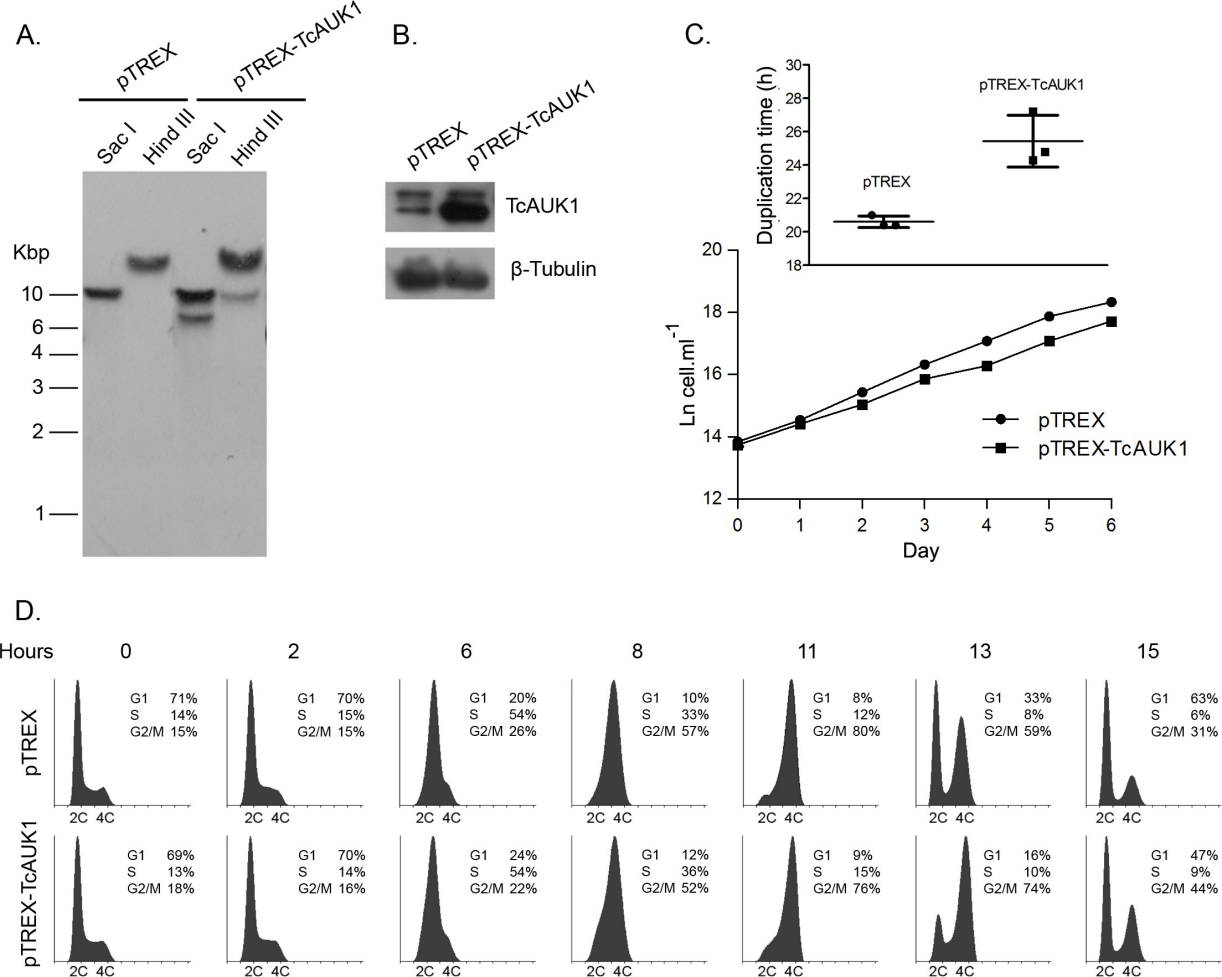
Analysis of TcAUK1 overexpressing epimastigotes. **(A)** Isolated DNA from pTREX and pTREX-TcAUK1 epimastigotes was treated with different restriction endonucleases and analyzed by Southern blot to confirm the extra copy of TcAUK1 in the genome of transgenic cells. **(B)** Immunoblot against TcAUK1 protein in pTREX and pTREX-TcAUK1 cells extracts. The β-Tubulin protein was used as loading control. **(C)** pTREX and clonal pTREX-TcAUK1 epimastigotes were cultivated and monitored for cell growth every day. Cell number was plotted in a logarithmic scale and the presented data is a mean ± s.d. of three independent cell cultures. Cells duplication time in each independent culture was calculated and a Paired T test (p<0,05) was performed to compare between pTREX and pTREX-TcAUK1 epimastigotes (inset). **(D)** Flow cytometry analysis of DNA content in pTREX and pTREX-TcAUK1 epimastigotes. Cells were synchronized at G1-S transition and cell cycle progression was monitored up to 15 hs after release.

The cell cycle stage dependent localization in epimastigotes of TcAUK1 is in accordance to the typical behavior of an Aurora B kinase, localizing inside the nucleus and appearing in association with the mitotic spindle during mitosis. Therefore, TcAUK1 could be fulfilling a possible role in spindle organization and chromosome segregation during cell division. The localization at each side of the kinetoplast during interphase is a novel observation that it has not been described in any other kinetoplastid. For this reason, to confirm this result with a different experimental approach, we generated epimastigote forms expressing a TcAUK1-GFP fusion protein by using the episomal, low-expression level vector pTEX. Fig 5B shows that TcAUK1-GFP presents a similar localization pattern to that observed by immunostaining. This novel localization could be reflecting roles of TcAUK1 in process other than nuclear division. In order to address the study of TcAUK1 possible roles, epimastigotes of *T*. *cruzi* overexpressing TcAUK1 were generated. Cells were transfected with the construction pTREX-TcAUK1 and stable overexpressing cells were selected under presence of G418 in the culture medium. After the selection period pTREX-TcAUK1 vector integration into the genomic DNA was confirmed by Southern blot analysis of the transfectant pool (Fig 6A). Parasites transfected with an empty pTREX vector were used as a control. Individual clones were isolated by the limiting dilution method, and a single clone in which TcAUK1 overexpression was confirmed by western blot was used for subsequent studies (Fig 6B). Considering that TcAUK1 seems to be involved in mitosis, we hypothesized its overexpression could alter cell cycle progression, modifying the culture growth rate. Three independent cultures of pTREX (control) and pTREX-TcAUK1 epimastigotes were initiated at 1x10^6^ cells.ml^-1^ and counted for cell density every 24 h for six consecutive days. Fig 6C shows the growth curve for pTREX-TcAUK1 and pTREX epimastigotes cultures, where it can be observed that pTREX-TcAUK1 epimastigotes had slightly lower growth rate. When cell duplication time (DT) was calculated based on the specific growth rate (μ) between days two to five in each culture (Fig 6C, inset), the mean of pTREX parasites was 20.6 (SEM 0.2) hours whereas for TcAUK1 overexpressing epimastigotes was 25.4 (SEM 0.895) hours (statistical significance was determined by Student's Paired *t*-test, P<0.05). In order to elucidate how cell cycle progression could be affected by TcAUK1 overexpression, hydroxyurea (HU) synchronized cultures were subjected to propidium iodide DNA staining followed by flow cytometry. For this, cultures of pTREX and pTREX-TcAUK1 cells were arrested at G1/S transition by incubating with hydroxyurea (HU). After this treatment cells were released and samples were taken every two hours for cell cycle analysis. Results depicted as histograms in Fig 6D show that both cultures progressed similarly through G1 and S phases (0 to 8 h after HU removal), but when cells entered to G2/M (11 h after HU removal) a delay was detected in TcAUK1 overexpressing cells when compared to pTREX epimastigotes. While at 13 h after-release from HU most of pTREX parasites completed mitosis (56% cells in G1 and 38% in G2/M), at this same time point pTREX-TcAUK1 parasites were still retained in G2/M phase (41% cells in G1 and 50% in G2/M). It was not still up to 14 hs post-release that most of pTREX-TcAUK1 cells complete mitosis (55% cells in G1 and 36% in G2/M), meaning that mitosis concretion in these cells was at least one hour retarded. Despite this observation, at 15 h after HU release, both cultures showed similar profiles in the cell cycle histogram. Thereby, the delay in the normal progression of the cell cycle in TcAUK1 overexpressing parasites supports the idea that TcAUK1 is involved in events occurring during cell division.

**Fig 7:**
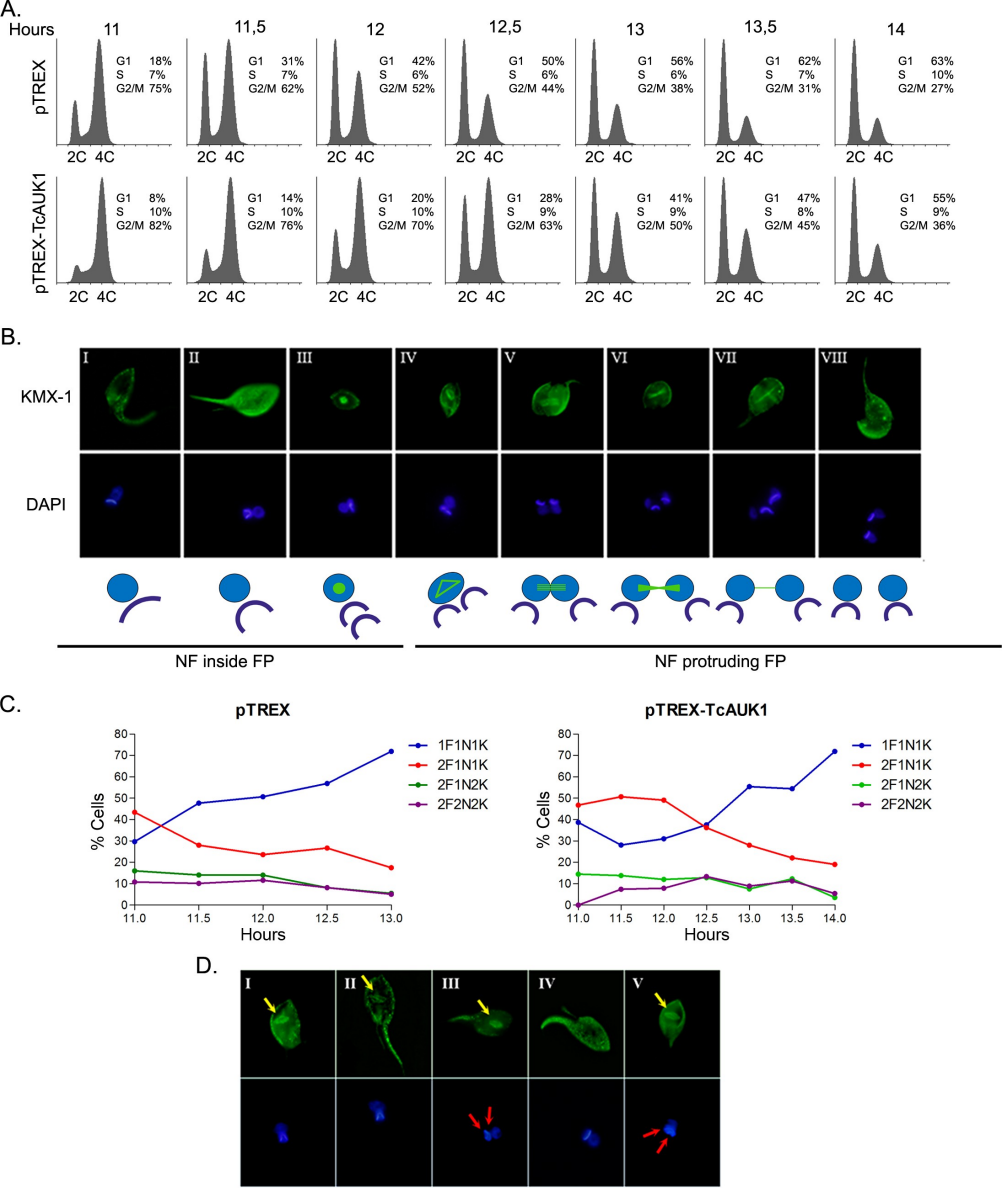
Effect of TcAUK1 overexpression on cell cycle progression in epimastigotes. **(A)** Mitotic progression of pTREX and pTREX-TcAUK1 synchronized cells was monitored by flow cytometry, measuring DNA content every 30 min between hours 11 to 14 post-releasing. **(B)** Mitotic spindle dynamics and DNA structure duplication (nucleus and kinetoplast) in WT cells were observed by immunostaining with mouse KMX-1 monoclonal antibody and DAPI dye, respectively. In the schematic representation of the events captured by microscopy: green is the mitotic spindle, blue the nucleus and dark blue the kinetoplast; NF means new flagellum and FP is flagellar pocket. **(C)** Graphical representation of cells with different number of flagellum (F), nucleus (N) and kinetoplast (K) in synchronized control (pTREX) and overexpression cells (pTREX-TcAUK1) at different time points after realizing (11 to 14 hs). Data are presented as the percentage of over 200 cells counted. **(D)** Mitotic spindle assembling (yellow arrows) and flagellum duplication in TcAUK1 overexpressing cells by immunostaining with mouse monoclonal KMX-1 antibody. Cell’s nucleus and kinetoplast (red arrows) were counterstained with DAPI.

A more detailed analysis of the progression through G2/M (11 h to 14 h after HU removal) of synchronized control and TcAUK1 overexpressing cultures was performed (Fig 7A).

Considering the limited information available on *T*. *cruzi* mitosis and the wide differences between lab strains, we initially set to analyze the dynamic of organelle duplication in WT epimastigotes. Progression through G2/M is characterized by the organized and timed duplication of 1- the flagellum, 2- the kinetoplast and 3- the nucleus. According to our observations, once the new flagellum is synthesized and protrudes from the flagellar pocket, the kinetoplast starts to duplicate, first increasing its length (Fig 7B, panel I DAPI) and then acquiring a “V” shape with the concave side facing the base of the flagella (Fig 7B, panel II DAPI). After that the kinetoplast “breaks” giving rise to the two daughter kinetoplasts (Fig 7B, panel III DAPI), that localize one behind each other (anteroposterior arrangement). Concurrently, the mitotic spindle starts to form in the nucleus, observed as a circular accumulation of β-tubulin (Fig 7B, panel III KMX-1). Next, while the new flagellum continues increasing its length, both kinetoplasts adopt a side by side configuration (lateral arrangement) and the mitotic spindle assembling begins (polygonal structure) in the nucleus (Fig 7B, panel IV KMX-1 and DAPI). This last event marks the end of the G2 phase and the beginning of mitosis (M). During this phase, the spindle elongates while the nucleus is divided in two (Fig 7B, panel V KMX-1 and DAPI). At the end of the nuclear division, sister nuclei and kinetoplasts continue to migrate in opposite direction while cytokinesis begins with the cleavage furrow formation in the anterior edge of the cell (Fig 7B, panel VI, VII and VIII KMX-1 and DAPI). As a result of this in-depth analysis of G2/M progression, two specific structural characteristics can be considered as landmarks: one is the length of the new flagellum in 2F1N1K cells and the other is the relative localization of the two kinetoplasts in 2F1N2K cells.

The microscopic analysis of the above described events in pTREX and pTREX-TcAUK1 synchronized epimastigotes between 11 h and 14 h after HU removal showed that entry into mitosis was delayed in TcAUK1 overexpressing parasites when compared to control cultures (Fig 7C). This is concluded from the observation that at 11.5 h after HU removal, control cultures showed a significant rise in the 1F1K1N population, indicating that most epimastigotes have undergone cell division. However, at this time point, most parasites in the overexpressing culture corresponded to the 2F1K1N subpopulation, with an augmentation of the 1F1K1N population only after the 12.5 h time point. The number of epimastigotes corresponding to the mitotic configurations 2F2K1N and 2F2K2N remains low throughout the analyzed period both in pTREX and pTREX-TcAUK1 cultures, indicating that this process occurs within a short time span. Moreover, this observation indicates that TcAUK1 overexpression does not alter mitotic events, in which case accumulation of the previously mentioned mitotic configurations would have been observed.

With the aim to confirm the effect of TcAUK1 overexpression on kinetoplast replication, we defined a method that allowed us to time this event with considerable accuracy. As described above, the new flagellum protrusion from the flagellar pocket and the beginning of mitotic spindle assembling (polygonal structure) constitute two independent events that indicate the start and the end of kinetoplast replication, respectively. Considering this, 2F1N1K cells were analyzed for kinetoplast division and new flagellum length, while 2F1N2K cells were examined for mitotic spindle formation and the lateral arrangement of duplicated kinetoplasts. In pTREX epimastigotes, at every time point all cells that displayed the new flagellum protruding from the flagellar pocket also presented kinetoplasts in the process of duplication. However, in pTREX-TcAUK1 cultures, cells bearing a long new flagellum outside the flagellar pocket but with a kinetoplast that had not entered the duplication process could be observed (Fig 7D, panel IV). Indeed at 12.5 h post-release, out of 53 2F1N1K cells, 47 had a protruding flagellum but only 22 showed a dividing kinetoplast. This structural configuration was detected in parasites from TcAUK1 overexpressing cultures obtained at every time point (from 11 to 14 h post release). Cells with 2F1N2K configuration were evaluated for the kinetoplasts arrangement (posterior or lateral) as well as presence or absence of mitotic spindle polygonal structure. The total population of pTREX epimastigotes examined showed the presence of a polygonal structure of the spindle as well as a lateral arrange of the kinetoplasts, as expected according to what was described for WT cells. However, when 2F1N2K TcAUK1 overexpressing epimastigotes were analyzed, 50% of the cells that showed the polygonal structure of the mitotic spindle displayed kinetoplasts still placed in an anteroposterior arrangement at 12.5 h after HU was removed (Fig 7D, panels III and V). This phenotype of 2F1N2K could observed at every time-point in pTREX-TcAUK1 epimastigotes. Moreover, an extreme phenotype of 2F1N1K cells with the polygonal structure of the mitotic spindle but a V-shaped kinetoplast was detected (Fig 7D, panels I and II) in epimastigotes overexpressing TcAUK1.

**Fig 8:**
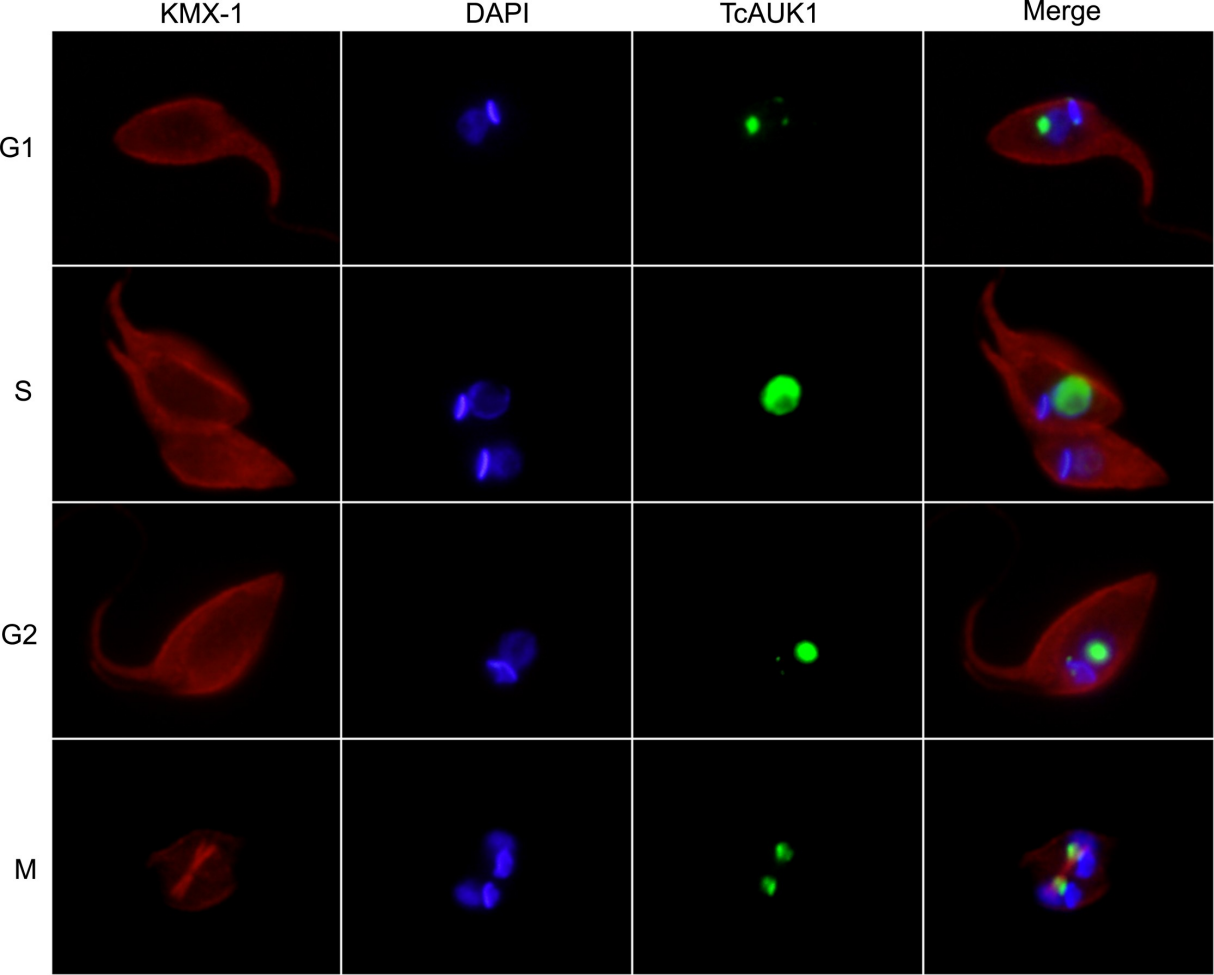
TcAUK1 localization in synchronized overexpressing epimastigotes. TcAUK1 overexpressing epimastigotes, at different phases of cell cycle, were stained for mitotic spindle (mouse monoclonal KMX-1 antibody), TcAUK1 (rabbit antiserum to TcAUK1) and nucleus/kinetoplast (DAPI).

Taking into account that the function of Aurora kinases is closely related to their localization through the cell cycle, we next studied TcAUK1 dynamics in overexpressing epimastigotes during the different phases of cell cycle, considering kinetoplast and nucleus duplication and mitotic spindle assembly as hallmarks of this process. Interestingly, TcAUK1 in overexpressing cells was present almost exclusively in the nucleus during the entire cell cycle (Fig 8). This result differs from what was observed in WT epimastigotes, in which TcAUK1 localizes in both extremes of the kinetoplast during the interphase (G1 and S phases) and only is detected in the nucleus just before mitosis begins (Fig 5). This observation led us to hypothesize that, when overexpressed TcAUK1 loses its localization at the kinetoplast and this could be affecting replication process of this organelle.

## Discussion

In this work we identified three Aurora kinase genes present in *T*. *cruzi* genome: TcAUK1, -2 and -3. By analysis of genomic sequences, we found that TcAUK2 has two different forms differing only in its amino acid sequences by 3%, including a 7-residue insertion in the longer form of the gene (TcAUK2L). By Southern blot and PFGE, we determined that TcAUK3 gene is represented by more than one copy per haploid genome. Moreover, cDNA sequencing shows that transcripts of these copies differ in their 5´-UTR. Considering all this data we postulate that TcAUKs family is not conformed only by 3 members, but 5 members form part of this group of genes. The presence of two different forms of TcAUK2 could indicate that these proteins have different and specific functions. In the case of TcAUK3, considering that gene expression is mainly regulated at the post-transcriptional level in trypanosomatids the differences in the 5´ UTR of the different transcripts identified for these genes could denote differential regulation in their mRNA stability or translation efficiencies, being therefore subjected to selective protein expression regulation. These findings propose TcAUK2 and TcAUK3 as interesting proteins to be studied on the near future. TcAUK1, which is present as a single copy gene, appears as the closest *T*. *cruzi* Aurora kinase to metazoans Aurora kinases, based on the phylogenetic analysis. Moreover, its orthologue in *T*. *brucei* (TbAUK1) has been reported as the protozoan counterpart of mammalian Aurora B kinase [23]. These led us to focus our efforts on the in-depth characterization of TcAUK1.

After confirming the expression of TcAUK1 in the amastigote, trypomastigote and epimastigote stages (Fig 3), we next aimed to study its localization during epimastigotes cell cycle. By immunofluorescence, we detected TcAUK1 at two discrete cell cycle stage-dependent subcellular localizations: during interphase, it is localized at the extremes of the kinetoplast while in mitosis it resides inside the nucleus, associated with the mitotic spindle (Fig 4A). Furthermore, we extended the localization analysis to trypomastigotes and amastigotes isolated from culture supernatant as well as in intracellular amastigotes. Although the result of the immunoblotting shows that this protein is expressed in trypomastigotes obtained from culture supernatants, TcAUK1 could not be detected in this form of the parasite by immunofluorescence. Nevertheless, when TcAUK1 was labeled in intracellular parasites we found trypomastigote forms that express this protein located in the nucleus (Fig 4C, 2 days after infection). Therefore, it seems that TcAUK1 could be expressed in trypomastigotes at specific times during the infection cycle, probably indicating its participation in specific cellular processes. It is important to highlight that despite the trypomastigote, amastigote and epimastigote are the three forms that have been more deeply studied, during the differentiation that mediates the passage between these forms, the parasite adopts different intermediate stages. This involves a plethora of morphological events occurring in a dynamic way until the cell reaches its final form. According to what we have observed in the trypomastigote form, it is possible to postulate that TcAUK1 could be expressed for a short period of time and participates in events taking place during cell differentiation. Furthermore, the hypothesis of a short-lived TcAUK1 could explain why it can be detected when techniques that evaluate a wide population are used (e.g. western blot) but its detection remains elusive when observing individual events (e.g. immunofluorescence). In amastigotes, similarly to what was observed for epimastigotes, TcAUK1 adopts two different locations. It is found inside the nucleus of intracellular amastigotes at two days after infection (Fig 4C) but it locates at the extremes of the kinetoplast in extracellular amastigotes and intracellular amastigotes at advanced days of infection (Fig 4B and 4C). The fact that TcAUK1 adopts the same nuclear location in trypomastigotes than in amastigotes at early days of infections is another observation that suggests that this protein could be involved in the differentiation process. On the other hand, in actively replicating intracellular amastigotes (advanced cellular infections) and the epimastigote replicative form, the location of TcAUK1 at the extremes of the kinetoplast could indicate that this protein is involved in cell division processes.

The role of TcAUK1 during cell division was studied in detail in the epimastigote form of *T*. *cruzi*. TcAUK1 localization alternates between kinetoplast extremes at interphase and the nucleus during mitosis. The arrangement that TcAUK1 adopts in the nucleus (Fig 4A, 2F2K2N cells) in close association with the mitotic spindle and migrating with segregating chromosomes strongly suggests that this protein is the orthologue of human Aurora B. The overexpression of TcAUK1 leads a delay in the duplication time of the epimastigote form, as observed in the growth curves of control and pTREX-TcAUK1 parasites (Fig 6C). The results of cell cycle analysis by flow cytometry on synchronized pTREX and pTRX-TcAUK1 cultures showed that in the later, the time employed to conclude mitosis is longer than in control epimastigotes (Figs 6D and 7A). This result confirms that TcAUK1 plays an important role in cell division. To further investigate this, we analyze organelle division dynamics during G2/M. The prevalence of 2F1K1N population at longer times after HU removal in TcAUK1 overexpressing cultures (Fig 7C) suggests that in these cells kinetoplast duplication is altered. By timing the sequential duplication of flagellum, kinetoplast and nucleus in WT cells (Fig 7B), we noticed that overexpression of TcAUK1 leads to a delay in the start of kinetoplast duplication (Fig 7D). Considering that the role of Aurora proteins is closely related to its localization, a possible explanation for the altered kinetoplast duplication is the fact that overexpressed TcAUK1 lost its location at the extremes of the kinetoplast during cell cycle interphase, as is detected in Fig 8. The subcellular location of TcAUK1 in WT epimastigotes during interphase detected by immunofluorescence is very similar to the localization of the so-called Antipodal sites–a nucleation of proteins that are involved in different kinetoplast processes–in Kinetoplastids [18,41]. Hence, it is very likely that TcAUK1 interacts with different targets that participate in kinetoplast division at these sites. Further experiments should be performed to confirm this hypothesis. It has been widely documented that Aurora kinase B in metazoans is associated with other proteins to conform the so-called CPC. More recently it was found that *T*. *brucei* AUK1 (TbAUK1) it is also associated with other proteins conforming a complex and that, like the CPC, this is crucial to guide TbAUK1 across different targets during cell division [25]. Nevertheless, the possible role of Aurora kinases in other cellular processes has not been explored yet. Here, we report for the first time that TcAUK1 is not only involved in mitosis but also in the duplication of the kinetoplast. It is well known that Aurora proteins cellular function is regulated by different post-translational modifications, such as protein phosphorylation and SUMOylation [42–45]. It is possible that TcAUK1 localization in the extremes of the kinetoplast is determined by its interaction with other proteins or by post-translational modifications, or a combination of these. The loss of its localization could be associated with the failure of one or both of these possibilities due to non-physiological expression levels. This hypothesis is supported by the observation that in western blot experiments, in whole cell extracts of pTREX-TcAUK1 epimastigotes only the lower molecular weight band increased its intensity, indicating that there are at least two different states for TcAUK1 (probably corresponding to different post-translationally modified proteins) but only one of them is augmented in overexpressing parasites (Fig 6B). If TcAUK1 needs to be post-translationally modified to interact with specific proteins responsible in order to be recruited to the kinetoplast extremes, then it could be possible that unmodified overexpressed TcAUK1 interacts and sequesters specific proteins, therefore impeding the modified TcAUK1 to reach the kinetoplast.

In summary, in this work we have identified the members of the Aurora kinase proteins in the protozoan *T*. *cruzi*. We have demonstrated that TcAUK1 protein behaves as the human Aurora kinase B regarding its role in nuclear division. Also, we have found that this protein is present in the three forms of the parasites life cycle and evidence shows that this protein could play a role in the differentiation between trypomastigote to amastigote forms. Interestingly, we have described a novel function for this protein in direct involvement in the initiation of kinetoplast duplication. This represents the first description of a role for Aurora kinases other than its widely studied function in mitosis. This stands as a valuable contribution in the attempt to understand in a molecular level the complex processes that take place during the life cycle of protozoan organisms.

## Supporting information

S1 Fig Sequence alignment of human Protein Kinase A and *T. cruzi* Aurora kinases. A multiple sequence alignment between the catalytic domain of human Protein Kinase A (*PKAc_alpha*) and the Aurora kinases from *T*. *cruzi* (*TcAUKs*) was performed by ClustalW algorithm. Conserved Glycine Rich loop and Activation Loop are highlighted (yellow squares). Also, are pointed residues involved in the kinase activity of *PKAc_alpha* and conserved in TcAUKs. Among them are the catalytic residues that adopt a spatial conformation required for enzyme activity. The Asp166 that conforms the dipeptide RD **(1)** together with Arg165 that interacts with the OH group of the substrate’s side chain. The Asn171 **(2)** that plays two relevant functions that allow enzyme activity. First, this residue orientates the catalytic Asp166 by a hydrogen bridge interaction; and second, together with Asp184 from the tripeptide DFG **(3)** required for the binding of the divalent cation involved in the nucleotide recognition. The Activation Loop begins with the highly conserved motive DFG and ends in the Glu208 residue, which is part of the APE **(7)** domain present in most of the kinase proteins. This loop has the capacity to undergo great conformational changes between the active and non-active states of the kinases. For this is critical the phosphorylation of the Activation Loop and particularly one residue adopts a central position, the Thr197 **(6)**. In the active conformation of the enzyme this residue is phosphorylated, here with Asp184 of the APE domain playing a critical role. Another important residue is the Lys72 **(4)**, that is correctly spatial orientated by an ionic interaction with Glu91 **(5)**, and binds the α and β phosphate groups of the ATP during the catalysis. Finally is the Arg280 **(8)** that stablishes a hydrogen bridge bound with the APE domain at the end of the Activation Loop.
(TIF)

S2 Fig Evolutionary tree constructed by the maximum parsimony method from a MSA of TcAUKs catalytic domains and aurora genes described for metazoans and protozoans. Hs *Homo sapiens*, Xl *Xenopus laevis*, Dm *Drosophila melanogaster*, Dd *Dictyostelium discoideum*, Ce *Caenorhabditis elegans*, Sp *Schizosaccharomyces pombe*, Sc *Saccharomyses cerevisiae*, Gl *Giardia lamblia*, Lm *Leishmania major*, Tb *Trypanosoma brucei*.
(TIF)
