# Supplementary figures and images for: Aurora kinase protein family in Trypanosoma cruzi: Novel role of an AUK-B homologue in kinetoplast replication
Source: PLoS Negl Trop Dis. 2019 Mar 21;13(3):e0007256. doi: 10.1371/journal.pntd.0007256 (PMC6445472; doi:10.1371/journal.pntd.0007256)

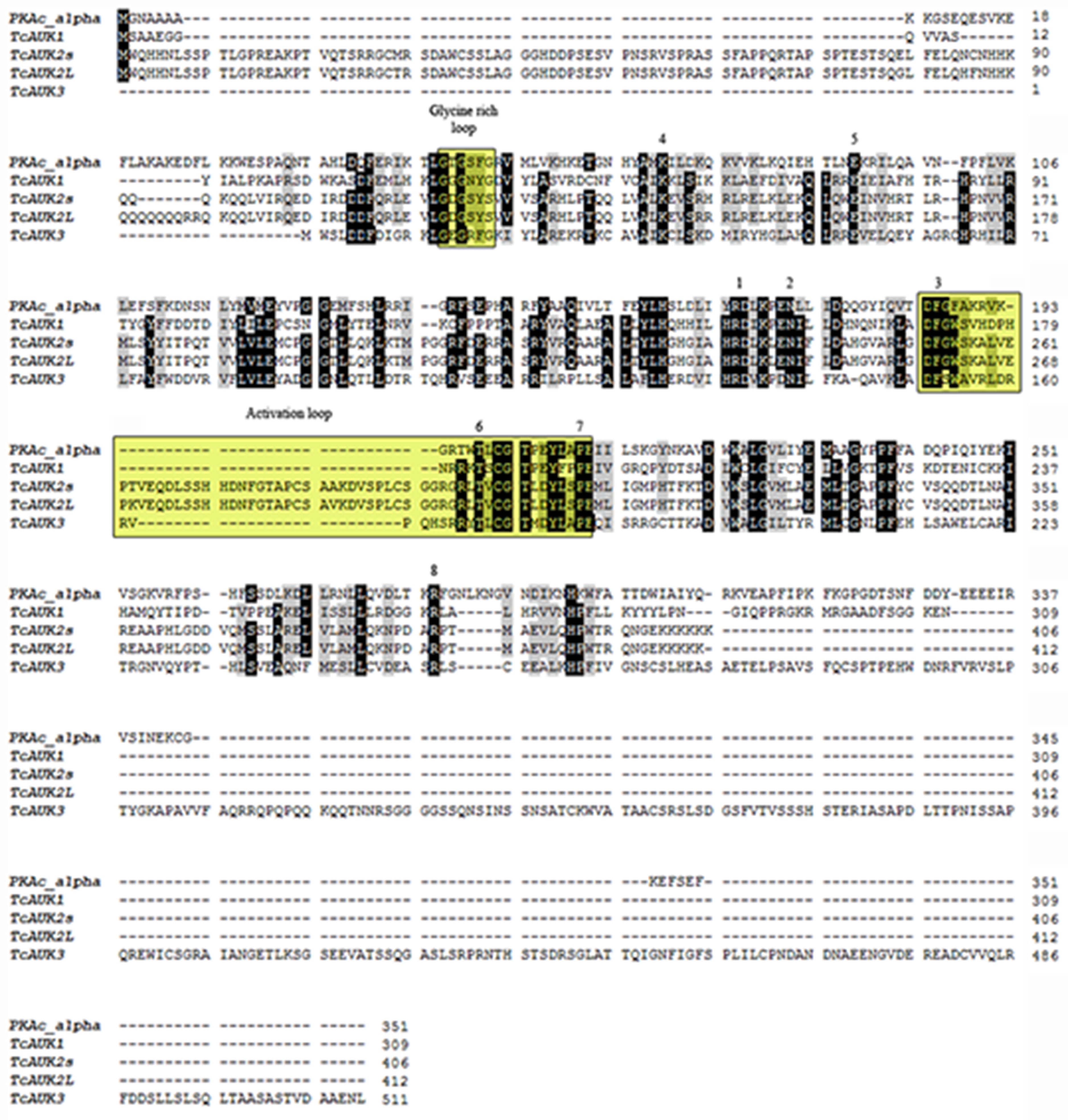

Supplement: S1 Fig — A multiple sequence alignment between the catalytic domain of human Protein Kinase A (PKAc_alpha) and the Aurora kinases from T. cruzi (TcAUKs) was performed by ClustalW algorithm. Conserved Glycine Rich loop and Activation Loop are highlighted (yellow squares). Also, are pointed residues involved in the kinase activity of PKAc_alpha and conserved in TcAUKs. Among them are the catalytic residues that adopt a spatial conformation required for enzyme activity. The Asp166 that conforms the dipeptide RD (1) together with Arg165 that interacts with the OH group of the substrate’s side chain. The Asn171 (2) that plays two relevant functions that allow enzyme activity. First, this residue orientates the catalytic Asp166 by a hydrogen bridge interaction; and second, together with Asp184 from the tripeptide DFG (3) required for the binding of the divalent cation involved in the nucleotide recognition. The Activation Loop begins with the highly conserved motive DFG and ends in the Glu208 residue, which is part of the APE (7) domain present in most of the kinase proteins. This loop has the capacity to undergo great conformational changes between the active and non-active states of the kinases. For this is critical the phosphorylation of the Activation Loop and particularly one residue adopts a central position, the Thr197 (6). In the active conformation of the enzyme this residue is phosphorylated, here with Asp184 of the APE domain playing a critical role. Another important residue is the Lys72 (4), that is correctly spatial orientated by an ionic interaction with Glu91 (5), and binds the α and β phosphate groups of the ATP during the catalysis. Finally is the Arg280 (8) that stablishes a hydrogen bridge bound with the APE domain at the end of the Activation Loop. (TIF) [file pntd.0007256.s001.tif]
